# Adopting a Framework for Rapid Real-World Data Analyses in Safety Signal Assessment

**DOI:** 10.1007/s43441-024-00694-7

**Published:** 2024-09-06

**Authors:** Lu Wang, Negar Golchin, Stephanie von Klot, Claudia A. Salinas, Katrin Manlik, Vaishali Patadia, Mary K. Miller, Julius Asubonteng, Rachel McDermott, Julie Barberio, Geoffrey Gipson

**Affiliations:** 1 Janssen Research and Development LLC, The Pharmaceutical Companies of Johnson & Johnson, 200 Tournament Drive, Horsham, PA 19044 USA; 2 BMS, New York, USA; 3 Boehringer Ingelheim International GmbH, Ingelheim am Rhein, Germany; 4 Eli Lilly & Co, Indianapolis, USA; 5 Bayer AG, Berlin, Germany; 6 Amgen, Thousand Oaks, USA; 7 Genentech, A Member of the Roche Group, South San Francisco, USA; 8 Regeneron, Tarrytown, USA; 9 Shionogi, Osaka, Japan; 10 Sanofi, Cambridge, MA USA

**Keywords:** Real-world data (RWD), Safety signal assessment, Pharmacovigilance, Transcelerate, Minimal protocol, Rapid data analysis

## Abstract

The expanding availability of real-world data (RWD) has led to an increase in both the interest and possibilities for using this information in postmarketing safety analyses and signal management. While there is enormous potential value from the safety insights generated through RWD, the analysis preparation, execution, and communication required to reliably deliver the evidence can be time consuming. Since the safety signal assessment process is a regulated and timebound process, any supporting RWD analyses require a rapid turnaround of well-designed and informative results. To address this challenge, a TransCelerate BioPharma working group was formed and developed a framework to help teams responsible for safety signal assessment overcome the challenges of working with RWD rapidly to deliver analyses within regulatory timelines. Here, a previously performed safety assessment was evaluated within the context of the developed framework to illustrate how the framework may be adopted in practice.

## Introduction

Pharmacovigilance, as defined by the World Health Organization, describes the activities related to the detection, assessment, understanding, and prevention of adverse drug events. Among these activities, the safety signal assessment process involves considering all available pharmacologic, clinical, and epidemiologic evidence to determine if a new risk is causally associated with a medicinal product or if a known risk has changed [1]. Traditional signal assessment evidence sources, such as clinical trial data, pharmacovigilance databases, and scientific literature, may sometimes be insufficient to confirm or refute a signal with complete confidence. Real-world data (RWD), typically collected during routine clinical care, can support safety signal assessment. However, methodological and operational challenges often impede completion of RWD studies within regulatory timelines.

TransCelerate BioPharma is a non-profit organization that aims to collaborate across the global biopharmaceutical community and improve the research and development of new therapies. In 2022, TransCelerate launched the Rapid Signal Assessment Using Real-world Data (RSA-RWD) Initiative to explore how safety signal assessment may be enhanced by “rapid RWD analyses,” defined as RWD analyses that can be conducted within required regulatory timelines. In its first year, the RSA-RWD Initiative conducted an extensive literature review and distributed a questionnaire to TransCelerate member companies to understand the safety signal assessment practice landscape and identify opportunities for rapid RWD analyses. Questionnaire responses revealed that key challenges to conducting RWD analyses rapidly include barriers to RWD access, the time-consuming nature of study planning and analysis, and the uncertainty of health authority acceptance of minimal or non-protocolized analysis approaches for signal assessment [2]. In response, the RSA-RWD Initiative developed a publicly available RSA-RWD Framework [3] to support safety management teams in conducting rapid RWD analyses while maintaining essential elements to deliver accurate, transparent, and value-adding analyses aligned with stakeholder expectations.

The objective of the present work was to demonstrate an application of the RSA-RWD Framework to the planning phase of a pharmacoepidemiologic RWD analysis, thus illustrating how the framework may be adopted in practice.

## Methods

### The RSA-RWD Framework

**Fig. 1:**
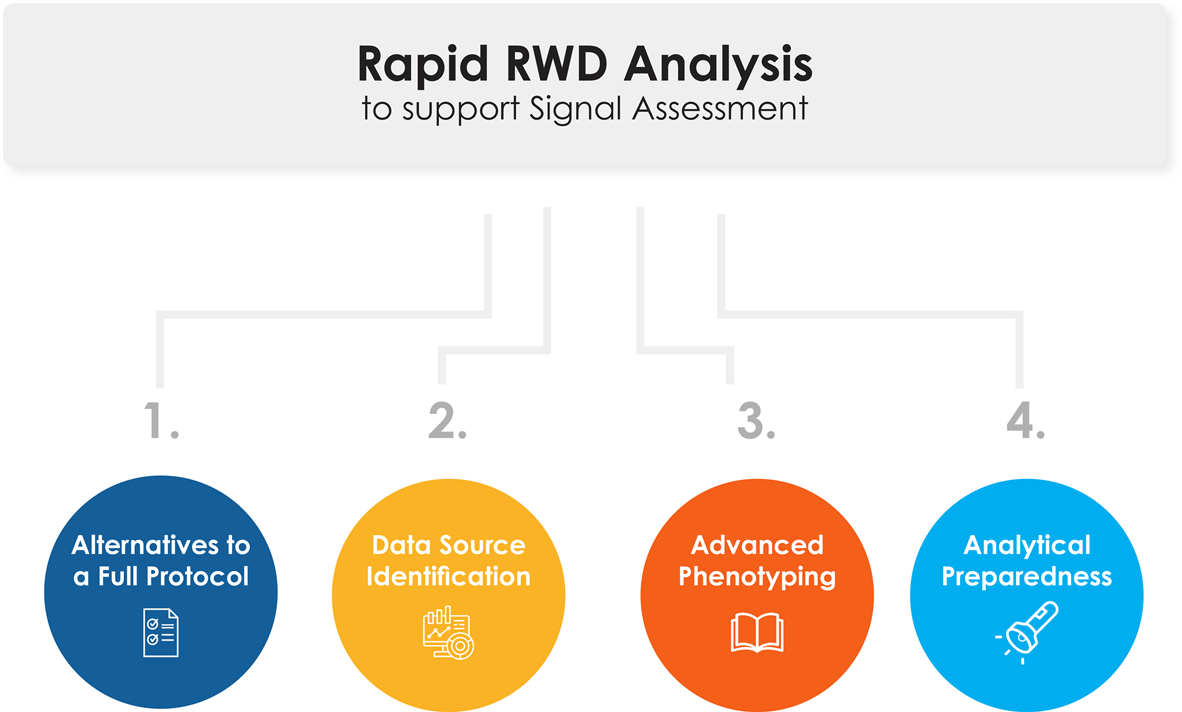
Four key considerations for conducting rapid RWD analysis for safety signal assessment

The publicly available RSA-RWD Framework was developed to address the key challenges in conducting rapid RWD analyses identified from the literature review and the responses to the TransCelerate member company questionnaire [3]. This framework provides an end-to-end, high-level process map to guide cross-functional safety management teams through several best practices for rapid RWD analysis in safety signal assessment. Specifically, the RSA-RWD Framework outlines four key considerations for rapidity (Fig. 1), which are centered on preparing or performing tasks in advance: (1) alternatives to a full protocol, (2) data source identification, (3) advanced phenotyping, and (4) analytical preparedness. For clarity, advanced phenotyping here means the proactive development of phenotypes - which are algorithms or definitions that “identify individuals who exhibit certain phenotypic traits, such as the same diseases, characteristics, or set of comorbidities” [4].

**Fig. 2:**
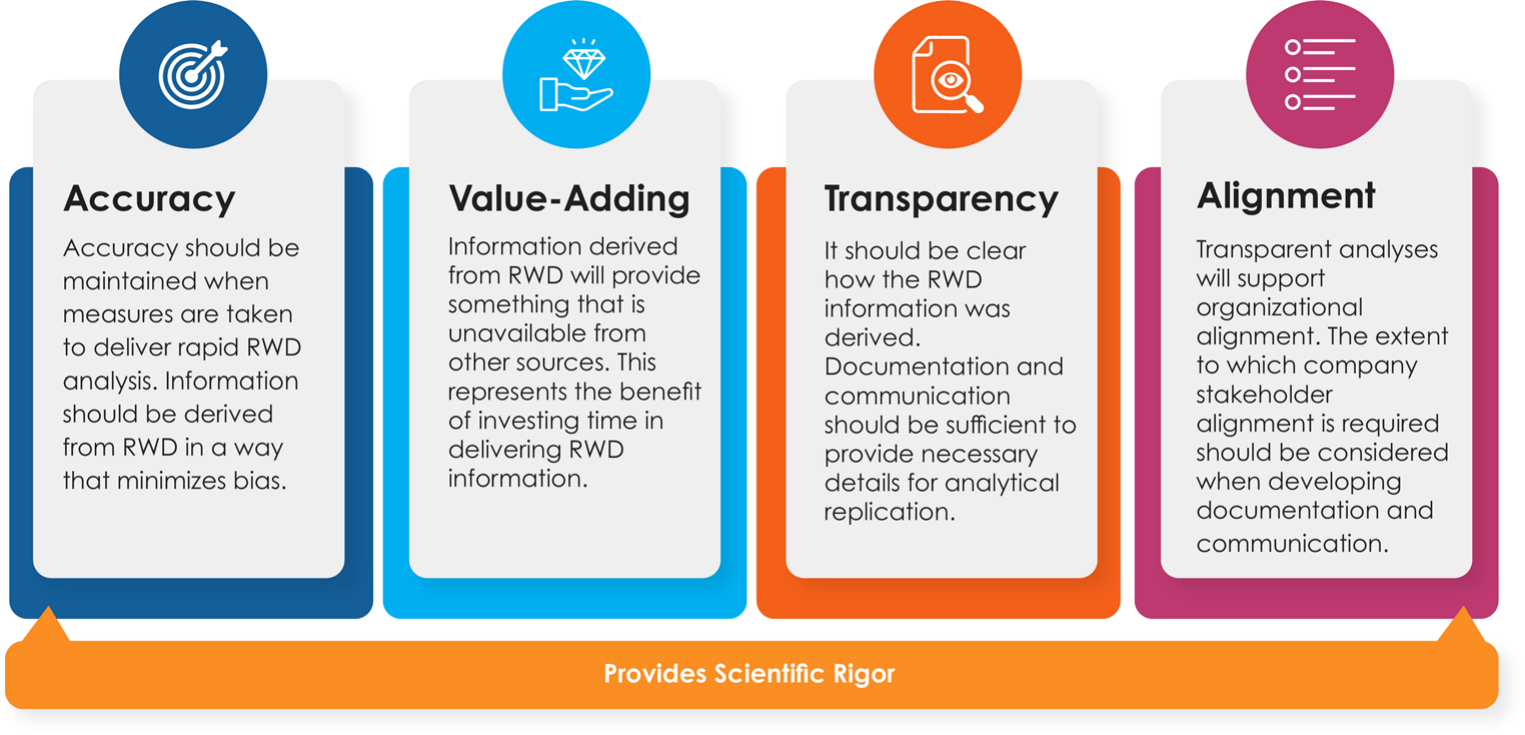
Four pillars for supporting scientific rigor when conducting rapid RWD analysis for safety signal assessment

In addition to these considerations developed by the Transcelerate team, we also explored insights from a concurrent effort, the International Council for Harmonisation of Technical Requirements for Pharmaceuticals for Human Use (ICH) Multidisciplinary Guideline 14 (M14) [5]. As a result of the review of the Guideline, we decided to add an additional consideration to the current evaluation: (5) conceptual to operational question mapping. To support scientific rigor when conducting rapid RWD analysis for safety signal assessment, the RSA-RWD Framework recommends upholding the four pillars of accuracy, value-adding, transparency, and alignment (Fig. 2) when addressing the key considerations for rapidity. Specifically, these opportunities for delivering rapid analyses without compromising scientific rigor will henceforth be referred to as “identified enablers.”

### Framework Application

**Fig. 3:**
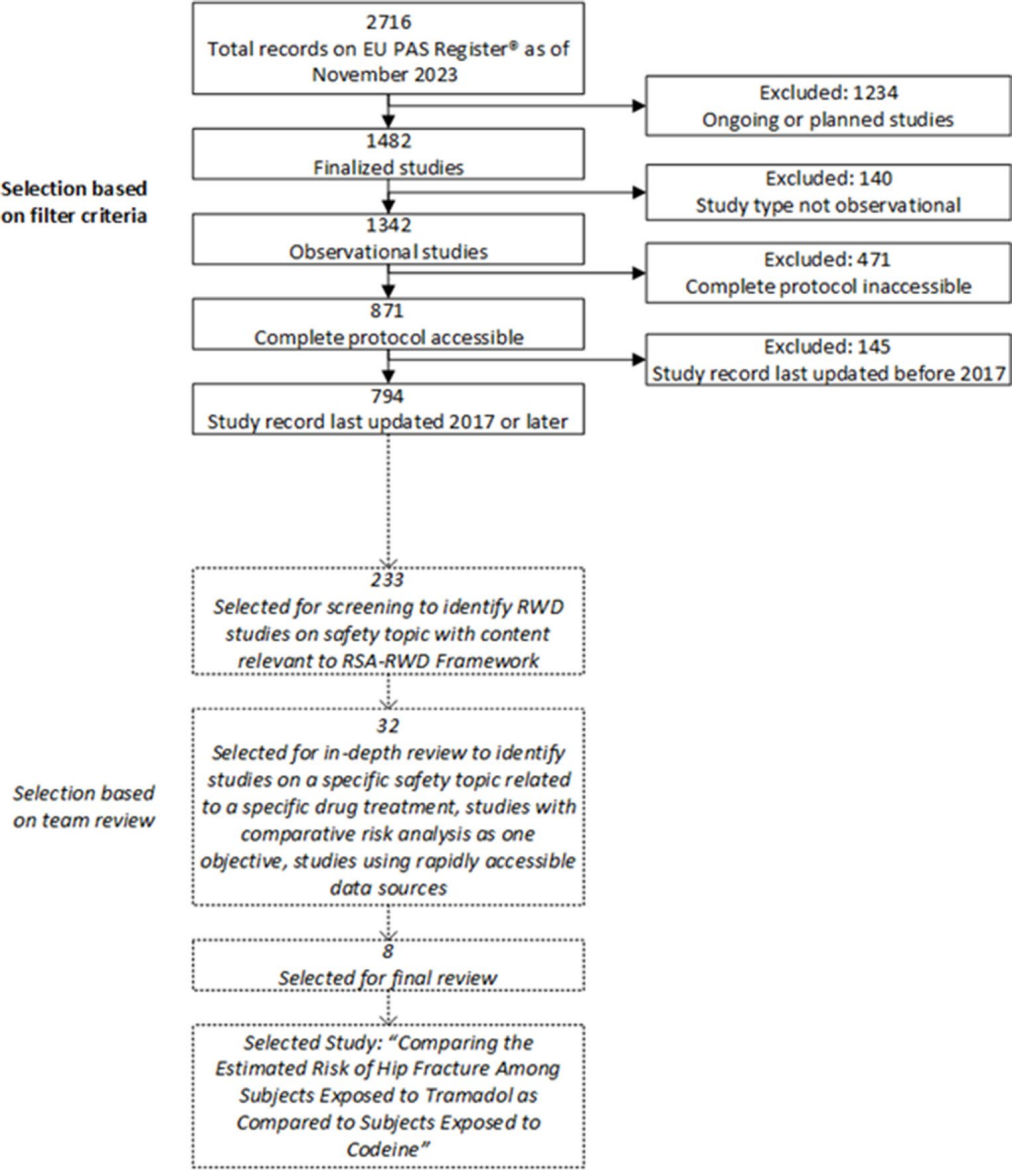
Stepwise approach for reviewing study records on the EU PAS Register® to select a single study protocol for demonstrating an application of the RSA-RWD Framework

To demonstrate a realistic application of the RSA-RWD Framework to support safety signal assessment, an observational study was selected from the publicly accessible European Union electronic Register of Post-Authorisation Studies (EU PAS Register®) [6]. The goal of selecting a preexisting study was to illustrate which RSA-RWD Framework considerations had been implemented by the study team and how incorporating the remaining considerations could have addressed the research question more rapidly. To identify a suitable study for this framework demonstration (Fig. 3), all EU PAS Register® records of recently finalized (2017 or later) observational studies with full protocols that were executed by TransCelerate member companies were considered. Candidates were screened to identify studies that addressed a drug safety topic using RWD and had content related to the RSA-RWD Framework considerations. Those that passed this initial screening were reviewed in-depth to identify studies that addressed a research question on a specific safety topic related to a specific treatment, had a comparative risk analysis as one objective, and used a rapidly accessible data source. The RSA-RWD Initiative working group reviewed the final candidates and voted on a preferred study. The selected study was then qualitatively evaluated against the identified enablers. Of note, although this approach provided an example study to support the current work’s aims, there were other candidates that could have served this purpose.

## Results

### Study Selected for Demonstration

The study selected from the EU PAS Register^®^ for the RSA-RWD Framework demonstration is titled, “Comparing the Estimated Risk of Hip Fracture Among Subjects Exposed to Tramadol as Compared to Subjects Exposed to Codeine” (EUPAS36038). Results of the completed study were published by Voss, et al. in 2022 [7]. The research plan was motivated by a previous publication by Wei, et al. (2020) [8] that reported an increased risk of hip fracture among new users of tramadol versus codeine. Voss, et al.’s study aimed to re-assess this relationship after addressing limitations of the prior publication (e.g., improved propensity score methods, use of morphine equivalents to estimate the exposure). The following sections provide a comprehensive assessment of the Voss, et al. study protocol against the RSA-RWD Framework. For each consideration for rapidity in the RSA-RWD Framework, we’ve outlined several identified enablers that were either implemented by the study team or could have allowed the research question to be addressed more rapidly.

### Consideration 1: Alternatives to a Full Protocol

Development of a fully specified and executable study protocol for safety signal assessment involves a complex process of document creation, methodological considerations, organizational alignment, and approval. As an opportunity for rapidity, the RSA-RWD Framework describes that a simplified, alternative RWD analysis plan [9] may be used.

### Identified Enabler 1: Use of an Abbreviated Specification

**Fig. 4:**
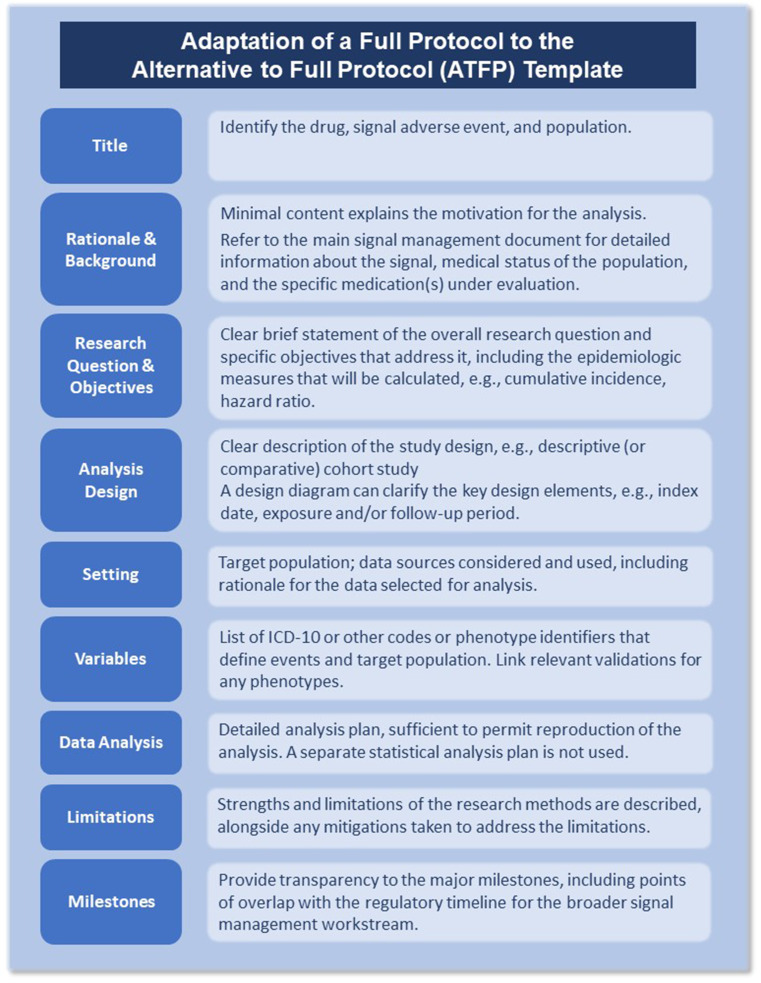
Summary of simplified protocol elements

The selected Voss, et al. study describes the key design elements and planned analyses using a full protocol consistent with the requirements set forth by the European Medicines Agency (EMA) (*Guideline on Good Pharmacovigilance Practices Module VIII* [10] *and Addendum I* [11]), Food and Drug Administration (FDA) (*Postmarketing Studies and Clinical Trials—Implementation of Sect. 505(o)(3) of the Federal Food, Drug, and Cosmetic Act* [12]), and the Pharmaceuticals and Medical Devices Agency (PMDA) (*Guidelines for the Conduct of Pharmacoepidemiological Studies in Drug Safety Assessment with Medical Information Databases* [13]). In Fig. 4, we highlight the critical components that would be described if the TransCelerate Alternative to a Full Protocol (ATFP) Template for rapid signal assessment (RSA) was implemented.

### Consideration 2: Data Source Identification

Identifying a fit-for-purpose RWD source for safety signal assessment involves considering whether the database includes variables essential to answer the research question and can be accessed within an expedited timeline. As an opportunity for rapidity, the RSA-RWD Framework describes that establishing a readily available RWD catalog with descriptions of accessible RWD sources can accelerate selection of appropriate data sources.

#### Identified Enabler 1: Compiling a Repository with Fit-for-Purpose Databases and Database Catalog

The selected study considered 6 RWD sources and ultimately chose 4 for analysis. Direct communication with the study investigator confirmed use of a pre-existing repository of in-licensed data sources. Among the total of 6 RWD sources considered, a single data source was selected to replicate the original publication. Outcome phenotype performance (i.e. sensitivity, specificity, positive and negative predictive value) was assessed in the additional 5 RWD sources and 3 of 5 data sources showed acceptable performance. The 3 sources with acceptable performance and the single source chosen for prior study replication were ultimately selected for further analysis. Compiling such a repository and a catalogue with detailed descriptions, metadata, and characteristics of each database as an overarching strategy allows investigators to quickly identify RWD sources, meet research needs and proactively avoid delays associated with assessing fitness for the purpose of new RWD sources.

#### Identified Enabler 2: Utilize Tools to Quickly Generate Feasibility Counts and Evaluate Data Sources

Consistent with the suggested rapidity best practices, the selected study assessed feasibility counts in all considered databases using a standard tool (CohortDiagnostics [14]). In addition, performance of the outcome phenotype was evaluated in each database using a tool (PheValuator [15]).

#### Identified Enabler 3: Prioritize RWD Data Sources in a Common Data Model

The study protocol indicated that all databases were standardized to the Observational Medical Outcomes Partnership (OMOP) Common Data Model (CDM), version 5.3 [16]. The OMOP CDM includes a standard representation of healthcare experiences and common vocabularies for coding clinical concepts, which enables rapid and consistent analysis across varied data sources.

### Consideration 3: Advanced Phenotyping

Planning an observational study for safety signal assessment must consider how to prioritize and validly define phenotypes of interest (e.g., adverse drug reactions) using administrative codes and algorithms in the chosen RWD source. As an opportunity for rapidity, the RSA-RWD Framework recommends that teams develop code lists and specify algorithms for commonly used phenotypes of interest in advance of conducting rapid RWD studies (i.e., “advanced phenotyping”).

#### Identified Enabler 1: Create or Utilize Existing Medical Definitions and Phenotype Libraries

The selected study’s protocol presented the code lists and algorithms used to define the target cohorts, comparator cohorts, covariates, and outcomes. It is unknown whether these definitions were pre-existing. Developing a library of definitions for various medical conditions allows for future rapid RWD analysis.

#### Identified Enabler 2: Implement RWD Data Source Catalogues or Dashboards and Code Lists for Phenotype Development

The selected study considered several data sources that were standardized to the OMOP CDM. Knowledge of the OMOP coding system and of the type of information available in each data source was required to create appropriate definitions for the medical conditions of interest. Having this information summarized in data source catalogs or dashboards can provide a foundation for rapidly developing phenotype definitions as needed for future RWD analyses.

#### Identified Enabler 3: Prioritize the Phenotypes that are More Commonly Investigated

The outcome of interest in the selected study, hip fracture, is a frequently observed health issue, especially in elderly populations. Such commonly observed clinically significant conditions could be prioritized in phenotype development. Archives of these prioritized phenotypes can become a rich resource of pre-existing phenotypes for accelerated RWD analysis.

### Consideration 4: Analytical Preparedness

Designing a high-quality RWD analysis plan for a safety signal assessment requires several decisions to be made related to patient characterization, comparator cohort definitions, measurement of exposure time, methods to control for confounding, and outcome assessment. As an opportunity for rapidity, the RSA-RWD Framework recommends that the creation or use of standard programming code, packages, and output templates can facilitate rapid results generation.

#### Identified Enabler 1: Pre-develop Standard Programming Codes

The selected study used a publicly available standard programming code, which enhanced efficiency and reduced potential for errors.

#### Identified Enabler 2: Establish Analytical Programs, Platforms, or Methodologies for RWD Analysis with Pre-specified Modules

The selected study used a pre-developed analytical platform equipped with methodologies tailored for RWD analysis (e.g., propensity scores, visualization tools).

#### Identified Enabler 3: Pre-develop Standard Output Templates

The protocol of the selected study described how the output will be organized into tables and figures however, specific output templates are not presented. Given that the described tables are common to RWD analyses (e.g., attrition, descriptive characteristics, covariate balance, event counts, incidence rates), developing standard templates for future analyses is an opportunity for rapidity.

### Consideration 5: Conceptual to Operational Question Mapping

The ICH M14 Guideline [5] outlines the differences between conceptual and operational definitions for exposures, outcomes, and confounders. The relative strengths, limitations, and uncertainties in applying an operational definition to closely approximate a conceptual definition and the need for validation are important considerations for all RWD analyses, whether rapidly executed or not. However, since rapid analyses will generally afford less opportunity for exhaustive consideration of these important topics, it is recommended that teams apply attention to the following identified enablers.

#### Identified Enabler 1: Validity of Data Collected in a Real-world Setting

The selected study analyzed RWD from administrative healthcare databases, which are collected during routine healthcare delivery rather than for research purposes. No standardized methodology was implemented to validate the recorded information. Discrepancies may exist between database records compared to true medical conditions or drug exposures (e.g., drug dispensing does not necessarily mean the medication was consumed). In the selected RWD study, because codeine-containing products can be purchased over the counter in the UK, drug exposure captured in the Clinical Practice Research Datalink (CPRD) database may not represent true exposure. In contrast, since these medications require prescription in the US, drug exposure captured in the US claims databases is more likely to be accurate and complete.

#### Identified Enabler 2: Operational Definition and Communication of Validation Efforts

The objective of the selected study was to assess whether exposure to tramadol, relative to codeine, causes a different risk of experiencing hip fracture in one year [7]. To achieve that goal, the investigators developed multiple operational definitions for the target cohort, comparator cohort, and outcome. The investigators not only replicated the operational definitions from a previously published study (Wei, et al., 2020) [8] but also used a modified approach to better represent conceptual definitions. The protocol specified all operational definitions and provided performance characteristics from validation of the outcome definitions. There are notable differences between the conceptual definitions in the original medical question and the operational definitions used in the two RWD studies (Wei, et al. and Voss, et al.) [7, 8], which have led to different results.

#### Identified Enabler 3: Translation of Evidence Generated from Operational Definitions

To allow translation of evidence generated from RWD analyses that have operationalized conceptual definitions, careful control for bias is essential. In the selected study, multiple analytical strategies were applied to improve comparability of the target and comparator cohorts and detect potential confounding in the risk estimate, including additional exclusion criteria, propensity scores matching, and negative control outcomes. Though residual bias cannot be completely ruled out, these measures significantly improved validity. Importantly, the investigators explicitly described limitations to their approach (e.g., new use definition) and unexpected findings (e.g., limited importance of morphine milligram equivalents conversion), which provides readers with appropriate context for interpreting the results.

## Discussion

As RWD studies can provide valuable context to the profile of a medicinal product, they are increasingly being used in regulatory decision-making. Numerous regulators and non-governmental groups have developed guidelines and recommendations for designing and conducting RWD studies [10–13]. However, as safety signal assessments are timebound, there is a growing need to identify best practices for implementing high-quality RWD analyses that can meet regulatory timelines. TransCelerate’s RSA-RWD Framework was developed to provide a process map and outline several considerations to rapidly generate high-quality, scientifically rigorous, and robust evidence from RWD to support safety signal assessment.

In the present work, we have demonstrated an application of the RSA-RWD Framework to a published pharmacoepidemiologic study to illustrate how the framework may be adopted in practice. Notably, a single comparative safety study (EUPAS36038) was selected for this exercise as a tangible example [7]. Notwithstanding, many other types of RWD studies reviewed during our study selection process could have also benefited from adopting the key considerations for rapidity, including but not limited to characterization of target patients, estimation of event incidence rates in background populations, and drug utilization studies.

The selected study provided an example of a RWD analysis that has already demonstrated many RSA-RWD Framework considerations. However, areas of opportunity were identified in the remaining considerations that would have allowed the study to be executed more rapidly. First, the selected study clearly specified the study objective, algorithms for cohort creation, and covariate code lists in the study protocol. The RSA-RWD Framework recommends that *alternatives to a full protocol (Consideration 1)* may be employed to document these elements in a briefer format, enabling rapidity. Second, the selected study consulted a pre-existing repository of in-licensed RWD sources already standardized to the OMOP CDM and used standardized tools to rapidly generate feasibility counts. To further accelerate the *data source identification (Consideration 2)* process, the RSA-RWD Framework suggests creation of catalogs that can be consulted to quickly identify RWD sources that meet research needs. Third, if *advanced phenotyping (Consideration 3)* was performed prior to study start through forward-thinking development of algorithms and code lists, the burden of real-time development of the study variables could have been lessened. Fourth, the selected study demonstrated *analytical preparedness (Consideration 4)* by using publicly available standard programming codes and an RWD analytic platform; preparation of output templates could have further facilitated rapid results generation. Lastly, we have also evaluated important aspects of *conceptual to operational question mapping (Consideration 5)*. Aligned with the ICH M14 guideline [5], the selected study has detailed the development and validation of operational definitions, implemented appropriate methodology to control for bias, and described the study limitations.

The successful use of the RSA-RWD framework requires an interdisciplinary approach and will likely include input from different functions (e.g. safety clinicians, epidemiologists, biostatisticians). Overall, the high-level considerations outlined in the RSA-RWD Framework and illustrated in this application may help cross-functional safety management teams generate timely, accurate, and valuable evidence using RWD to support safety decision-making. For optimal application of the framework, it is suggested that preparatory work is performed in advance as much as possible so that RWD analyses can be performed rapidly when needed. Specific recommendations include having rapidly accessible fit-for-purpose data sources pre-identified, employing an alternative protocol that uses a briefer format, establishing pre-developed phenotypes and standard analytical programs for common analyses, and developing pre-configured output templates. While these preparative actions may represent additional work at the outset, they can be expected to enhance the rapidity and reproducibility of future analyses. In taking these actions to help realize simplicity and brevity, it is paramount to uphold the essential pillars for scientific rigor, such as accuracy and transparency, so that the rapid RWD analysis can deliver added value that is aligned with stakeholder expectations.

It is important to note that not every consideration of the RSA-RWD Framework will be applicable to all future RWD analyses for safety signal assessment. For example, some safety questions will still necessitate a full protocol and, therefore, the considerations for using alternatives to a full protocol will not be relevant. Additionally, pre-development of phenotype for rare adverse events may not be feasible and the operational definitions may instead need to be developed on a case-by-case basis. Research groups may also encounter barriers to establishing RWD repositories due to limitations imposed by contractual obligations and costs. Furthermore, such fixed data source repositories are likely unable to address all future research questions of interest. Nevertheless, in many cases, the RSA-RWD Framework will provide many opportunities to assist with delivering RWD analyses for signal assessment with enhanced efficiency. Finally, settings other than drug safety signal management that also need to consider multiple evidence dimensions under urgent decision-making timelines (e.g., health technology assessment, public health situational analysis) could also benefit from the considerations outlined in the RSA-RWD Framework.

## Conclusion

There is a growing need to rapidly implement high-quality RWD analyses to generate accurate and valuable evidence to support drug safety decision-making. The RSA-RWD Framework addresses methodological, operational, and technical challenges that often impede use of RWD in safety signal assessment because of resource and time constraints. Implementing the principles of this RSA-RWD Framework may assist in communication with health authorities, help manage sponsor expectations, and increase confidence in rapid RWD analysis.

## Data Availability

No datasets were generated or analysed during the current study.
